# The Effect of Digestion and Digestibility on Allergenicity of Food

**DOI:** 10.3390/nu10091129

**Published:** 2018-08-21

**Authors:** Isabella Pali-Schöll, Eva Untersmayr, Martina Klems, Erika Jensen-Jarolim

**Affiliations:** 1 Comparative Medicine, The Interuniversity Messerli Research Institute of the University of Veterinary Medicine Vienna, Medical University Vienna and University Vienna, Veterinärplatz 1, 1210 Vienna, Austria; erika.jensen-jarolim@meduniwien.ac.at; 2 Institute of Pathophysiology and Allergy Research, Center of Pathophysiology, Infectiology and Immunology, Medical University of Vienna, Währinger Gürtel 18–20, 1090 Vienna, Austria; eva.untersmayr@meduniwien.ac.at (E.U.); martina.klems@meduniwien.ac.at (M.K.)

**Keywords:** anti-acid, acid suppressing medication, bariatric surgery, blocked digestion, food allergy, gastritis, impaired digestion, Maillard, reflux, ulcer

## Abstract

Food allergy prevalence numbers are still on the rise. Apart from environmental influences, dietary habits, food availability and life-style factors, medication could also play a role. For immune tolerance of food, several contributing factors ensure that dietary compounds are immunologically ignored and serve only as source for energy and nutrient supply. Functional digestion along the gastrointestinal tract is essential for the molecular breakdown and a prerequisite for appropriate uptake in the intestine. Digestion and digestibility of carbohydrates and proteins thus critically affect the risk of food allergy development. In this review, we highlight the influence of amylases, gastric acid- and trypsin-inhibitors, as well as of food processing in the context of food allergenicity.

## 1. Introduction

The prevalence of adverse reactions to food is still increasing. In the United States, an estimated rise from about 3% (1997–1999) to 6% (2016) of children younger than 18 years affected by food allergies has been reported [1]. A number of different factors are discussed to influence the development of food allergies. Among these factors are smoking incl. passive or second-hand smoke [2], the changed environment and/or pollution [3], altered vitamin D levels [4], and dual allergen exposure (skin contact with food proteins compared to oral exposure) [5]. Also, increased hygiene resulting in reduced microbiota diversity [6] or usage of antibiotics early in life disturbing the microbial balance in the intestine [7] seem to play a role. Furthermore, the diet of the mother during pregnancy/lactation [8], and additionally, the type and time point of complementary food introduction for the child could be important [9]. For the latter, the recommendations have been updated recently [10], now stating that introduction of allergenic food, e.g., peanuts, even to high-risk children should not be avoided or postponed [11,12,13].

Furthermore, digestion and digestibility could determine whether food proteins are tolerated or become sensitizing agents. This aspect has therefore even been taken up by the European Food Safety Agency in their scientific opinion about evaluation of allergenicity of food and feed proteins. Higher resistance to digestion or survival along the digestive tract seems to increase the sensitization capacity of a food component and renders it more immunogenic and/or allergenic. Based on this scientific background, the present review article highlights factors influencing protein digestion and digestibility.

## 2. Digestion of Carbohydrates: Amylase Action Critical for Starch Digestion and Microbiome

In green plants, starch accumulates as a product of photosynthesis. As a complex polysaccharide, it represents a significant compound of our diet and serves as energy supply, but also as food matrix. Also the food industry takes advantage of starch by supplementing it to infant food for maintaining “colonic health” [14]. Starch is digested by specific enzymes, i.e., amylases, which cleave the α-1,4-glucosidic bond of its major compound amylose, as well as the α-1,6-glucosidic bond of the second major constituent, amylopectin [15]. In microbes, the amylase enzyme group consists of 19 members, each with unique catalytic properties. They are technically applied in the starch saccharification industry [15]. However, transient malabsorption due to immaturity of the GIT during growth of the young child must be taken into consideration [16].

It is important to understand the biological impact of amylases, which are well conserved in the animal kingdom [17]. In humans, α-amylase is a product of the exocrine pancreas. Animal models suggest that microbial amylases could be supplied in pancreas insufficiency [18]. It is not known whether this will be linked to a risk for sensitization, but α-amylase per se when inhaled is a well-known occupational allergen. In baker’s asthma associated with the flour processing industry, allergenic amylase derives from contaminating fungi [19].

In mammals, amylase is also secreted into the saliva. Its role in starch digestion has been questioned due to its low amount relative to the overall amylase activity [20]. However, in vitro studies strongly propose that salivary amylolytic activity hydrolyzes up to 80% of bread starch in the first 30 min of gastric digestion, independent of acidification by the gastric juices [21]. This critically affects the quality of remnants reaching the intestine, which will affect the composition of the microflora (discussed below).

While in human medicine this is less known, psychologists take advantage of salivary amylase as a non-invasive biomarker for the evaluation of acute stress response [22] and it is increasingly used in behavioral medicine [23]. The biological relevance of this phenomenon might be a need of quick energy supply in form of glucose in the “fight-or-flight” reaction. Biomarker research indicates that stress also has an effect on immune reactions. For instance, the release of salivary α-amylase indicated that experimental stress was higher in rural participants raised in the presence of animals [24]. Acute or chronic stress may therefore quantitatively regulate amylase activity, and thereby impact on the composition of digested carbohydrates and subsequently affect microbiota composition (discussed below).

The amylase action on rapidly digestible starch (RDS) renders smaller products, like disaccharides and trisaccharides [25]. These are then further hydrolyzed to glucose by other enzymes, such as α-glucosidase in the small intestine [26]. However, both amylase and α-glucosidase may act synergistically. Some compounds represent slow-digestible starch (SDS), or resistant starch (RS) as larger leftovers, which persist the gastrointestinal transit to a large degree. Usually, resulting levels of malto-oligosaccharide indicate the degree of granular starch breakdown.

The starch breakdown by amylases is largely influenced by the composition of the food processing and matrix composition. Cooking has been shown to enhance the amylase breakdown of starch [27], which also depended on the individual α-amylase activity. Flavonoids are important plant constituents, which interfere with amylase activity by hydrophobic interaction in the food matrix or by formation of covalent bonds during cooking or in gastric juice, and therefore impair starch digestion [28]. This opens up potential intervention strategies in diabetic patients to decrease the fermentation speed of starch and thereby inhibit an undesired fast release of glucose. Starch may also form complexes with lipids in the food matrix, e.g., complex formation with palm oil interfered with the digestion of rice starches [29]. Interestingly, some fresh food may neutralize amylases by proteolysis. Kiwi contains actinidin, a cysteine proteinase, which specifically attacks amylase and thereby may inhibit starch digestion [30]. This may affect the presentation of allergenic epitopes in the food matrix.

Amylase in the duodenum also plays a key role in the breakdown of gluten and may therefore modulate its pathophysiologic role in celiac disease [31]. While starch forms complexes with gluten during baking of bread, amylase resolves them and makes gluten accessible for thorough protein digestion. Wheat on the other hand contains anti-enzymes, such as the ATIs (amylase-trypsin inhibitors) with a role in non-celiac gluten sensitivity (NCGS) [32]. Nutritional ATIs additionally stimulate the innate immune reaction via TLR4 [32] and thereby exacerbate allergic inflammation not only in the intestine, but also in the airways in mouse models [33,34]. It is hypothesized that industrial food processing contributes to the increased numbers of non-celiac gluten/wheat sensitivity by stabilizing e.g., starch-gluten complexes, thereby bypassing the salivary and pancreatic enzymes, leaving the digestion to mucosal amylases [35].

Processing may also affect the nanostructure of food, again affecting the amylase fermentation and hydrolyzed products thereof. Depending on the composition, the RS fraction can serve as a form of “prebiotics” fostering a bacterial community with benefits for health [36,37], confirmed recently in an animal model [38]. Dietary inclusion of RS changed the 16S rRNA profiles of the gene bacterial community, the profile of short-chain fatty acids (SCFA) and the overall lipid metabolism in pigs [39]. In humans, a high RS proportion resulted in a beneficial increase in the ratio of Firmicutes to Bacteroidetes [40], in favor of immune protection against allergies [41]. Therefore, starch digestion via modulating microbiota richness also impacts food allergy.

Overall, starch is a major nutrient compound and food matrix, and industrial processing critically interferes with its fermentation by amylase. Physiologically, stress enhances salivary amylase release, and pancreatic disorders are associated with polysaccharide maldigestion. Both, starch and amylase activity have implication for energy supply and the composition of RS remnants, which again critically affect microbiota composition.

Extracting the evidence from all aspects of pathophysiological starch digestion in correlation with life-style factors, we anticipate that amylase action may have an impact on the allergenicity of food by several means:

(1) It may result in epitope modification of plant food allergens or reveal neo-epitopes;
(2) Starch and other food matrix compounds may form stable complexes during food processing, supporting the transit of intact allergens;
(3) Amylase action affects the composition of fermentation products with significant effect on microbiota composition.

Presumably, this has impact not only on the control of celiac and non-celiac gluten hypersensitivity [32], but also on type I food allergy with early life being critical [42]. More studies need to be done to understand how exactly the microbiome could be manipulated in allergy and asthma [43], but targeting starch digestion could be an interesting option.

## 3. Digestion and Digestibility of Proteins Associated with Lipids or Carbohydrates

There is general agreement that resistance of proteins to gastric digestion is an indicator for potential allergenicity. For instance, in an vivo rat model digested vs. non-digested BLG was compared, and clearly the intact BLG induced more IgE, IgA, and IgG1, linking the digestion and digestibility of BLG directly to allergenicity [44]. This implies that any condition that keeps a certain protein intact adds to the risk of food allergy induction.

A very important family among allergens are lipid-transfer proteins (LTPs). It was recently shown that ligand binding can have different effects on their in vitro digestibility. In most cases, binding of lipids to LTPs increases resistance to digestion. This was for instance shown for LTP from peach and sunflower [45]. Sunflower seed was reported to be the most frequent elicitor of severe allergic reactions in Europe, even more frequent than peanut, and listed in the middle field of food sensitizations in European adults [46]. The LTP is stabilized against gastric digestion when phosphatidylcholine (PC) was added in vitro. However, in vivo proof that PC-stabilization also leads to increased allergenicity of the LTP so far is missing. Furthermore, the influence of (impaired) gastric vs. duodenal/intestinal digestion needs to be investigated for oral sensitization in animal models.

Furthermore, binding of lipids like PC to β-lactoglobulin and α-lactalbumin interferes with their digestibility [47,48]. However, sensitization studies, which directly compare allergen with or without attached lipid in vivo, are lacking.

Peptic digestion of grape LTP was not influenced by the presence of PC, and both molecules (with or without PC) were able to induce skin prick test reactivity in allergic patients. It seems important that the grape LTP is very stable to pepsin digestion, with or without the presence of lipid.

Similarly, binding of linoleic acid to wheat LTP did not change the gastric digestibility, and only slightly increased its susceptibility to gastroduodenal digestion via changes in the structure [49]. Wheat LTP was described as an important protein recognized by patients with food allergy to wheat [50], and in around 60% of people with baker’s asthma, although it elicits sensitization via the respiratory tract, where digestion might not play a crucial role [51].

In addition to individual molecules such as lipids the overall food matrix may play a crucial role in the availability of different proteins for enzymatic breakdown, as was shown e.g., for peanut allergens [52].

Apart from loading with different molecules and additional effects of food matrix, food processing may change the digestibility and allergenicity of food [53]. Pasteurization of milk is very common and important. However, this heating process can cause aggregation of food proteins such as β-lactoglobulin and α-lactalbumin. This aggregation was shown to enhance the uptake by Peyer’s patches and Th2-mediated antibody and cytokine production in mice [54].

During the heating of food products, which contain sugars and proteins, not only does aggregation of proteins occur, but so does the so-called Maillard reaction (MR). This reaction leads to products that are responsible for color, flavor and taste e.g., in many fast food products, bakery products or roasted peanuts. During this non-enzymatic reaction, free amino groups (mainly lysine and arginine) of protein side chains can be occupied by covalent binding of reducing sugars, i.e., glycation. Schiff bases are formed, followed by Amadori rearrangement and oxidative processes, altogether responsible for the formation of advanced glycation end products (AGE), a chemically heterogeneous and unstable group of molecules. These processes lead to a modified availability of enzymatic cleavage sites of the protein. Higher glycation of β-lactoglobulin resulted in reduced susceptibility to digestion by trypsin/chymotrypsin [55]. Glycation also decreased in vitro digestibility for patatin from potato [56], tropomyosin from scallop [57], and high-molecular weight peanut proteins such as Ara h 1 [58]. The peanut proteins were more resistant to digestion in the fried and roasted peanuts than in the raw and boiled samples. Also, wheat flour proteins in bread crumb and crust gained higher resistance and IgE-binding capacity during the baking process [59], as did the glycated egg protein ovalbumin, but not ovomucoid [60]. The latter study also showed a time-dependency during formation of MP, as OVA glycated for 96 h was much more stable to gastric and duodenal digestion than OVA glycated for 48 h.

Contrasting to these results with OVA, walnut 11S globulin after heating/roasting [61], and lysozyme and codfish parvalbumin after glycation [62,63] ended up in higher solubility and digestibility. These divergent observations may be explained by the different internal structural characteristics of the proteins, and additionally by different types of sugar used in the MR: galactose-glycation of β-lactoglobulin resulted in higher digestion-resistance than glycation with tagatose [64].

The AGE per se can also be found in plasma and urine correlating to the amount in the diet [65]. They can also be used by human microbiota of the lower gastrointestinal tract as energy source [66], and probably modify the microbiota composition. The appearance in the GIT and blood system makes AGE also likely to interact with immune cells, for instance activation of DC via AGE-receptors (AGE-receptor complex, scavenger receptors A and B, mannose receptor, CD36; reviewed in Ref. [67]) was shown [68]. Binding of roasted Ara h 1 was shown to occur to scavenger receptor CD36 and receptor for advanced glycated end products RAGE [69,70], and for roasted Ara h 3 to mannose receptor [71]. Most importantly, this engagement leads to cellular signaling resulting in pro-inflammatory responses and enhanced allergic sensitization, as was shown in a mouse model comparing raw vs. roasted peanut [69]. The animals sensitized with dry-roasted peanut extract showed higher IL-4, IL-5, and IL-13 levels, as well as more specific IgG- and IgE-antibodies and degranulation of effector cells. The usage of AGE-modified OVA (compared to native OVA) proved that the NFκB-pathway of DCs is involved in this outcome, as well as more efficient activation of OVA-specific CD4+ T-cells, releasing more Th2-specific cytokines like IL-4, IL-5 and IL-6 [72,73]. Overall, AGEs and more specifically glycated food allergens may have enhanced T-cell activation potential and thereby could increase the risk for allergic sensitization and/or effector cell reactions (reviewed in [74]).

In vivo data gathered with Maillard products and the effect on allergenicity are scarce. In humans, a diet rich in MR-products (MRP), like AGE, limited the digestion of the protein. This was shown in healthy young males as appearance of higher fecal nitrogen, lower absorbed nitrogen, and lower digestibility of nitrogen [75]. Animal models show different in vivo effects of MRP-application regarding the allergenicity. Depending on the conditions used, the capacity of the protein to evoke sensitization and/or allergic reactions in Balb/c mice increased for AGE-OVA [76] and for roasted peanuts [69]. In contrast, there was a decrease in sensitization potential for glycated tropomyosin and arginine kinase from crustaceans [77], for buckwheat allergen Fag e 1 [78], and for chickpea protein [79].

Finally, Maillard products also display an altered recognition by specific IgE present in allergic patients or animals. This might be due to (i) the changes in the tertiary and secondary structure, which can disrupt conformational or linear epitopes and reduce IgE-binding [80], (ii) formation of aggregates, which show enhanced degranulation [81], and (iii) formation of new IgE-epitopes, as was shown for Pecan nut, wheat flour and soybean. These foods only induced allergic reactions after cooking, long storage or heating [82,83]. Whereas important allergens from cherry (Pru av 1) [84], hazelnut (Cor a 1) [85], and milk (β-lactoglobulin) [86] showed reduced IgE-binding after heating in presence of (poly)saccharides, the allergens from peanut (Ara h 1 and Ara h 2) displayed significantly higher IgE-binding after non-enzymatic browning [87].

Importantly, it is necessary to define the final allergenicity of roasted food in vivo, as among 17 hazelnut-allergic patients, 5 still had positive DBPCFC-reactions, even though other methods (SPT, HR, specific IgE-binding) showed a reduced allergenicity of the roasted form of hazelnut [88].

Taken together, the heating of foods which contain reducing sugars together with proteins leads to the Maillard reaction and changes the conformation of the protein. This process can lead to (i) different digestibility of some proteins, (ii) masking of existing antibody epitopes, or (iii) formation of novel molecules, and may thereby also modify the immunogenicity and allergenicity of food proteins (reviewed in [74,89]). The resulting immunoreactivity of glycated proteins may decrease, remain unchanged, or even increase after food glycation [67].

With certainty, further studies are warranted to show the effects of the Maillard reaction for individual, structural diverse protein molecules, different sugars, the dependency on temperature, pH, duration of processing, water content and activity of the product, and probably also the food matrix. The effect on digestibility and subsequent immunogenicity and allergenicity has to be shown in vivo.

## 4. Digestion of Proteins: Gastric Acid is Critical for Adequate Protein Digestion and Prevention of Food Allergy

The digestibility of antigens has since long been considered a critical prerequisite for the induction of food allergy [90]. However, also a number of digestion-labile proteins were shown to induce allergic symptoms by primary sensitization without any co-existing pollen allergy, for instance hazelnut [91].

Digestion of proteins -and therefore most food allergens- is initiated in the stomach. A low pH is essential for the inactive enzyme pepsinogen to get activated into pepsin [92]. However, if acid-suppressing drugs are given, the pH increases considerably (e.g., up to 5 with proton pump inhibitors, PPI).

**Figure 1:**
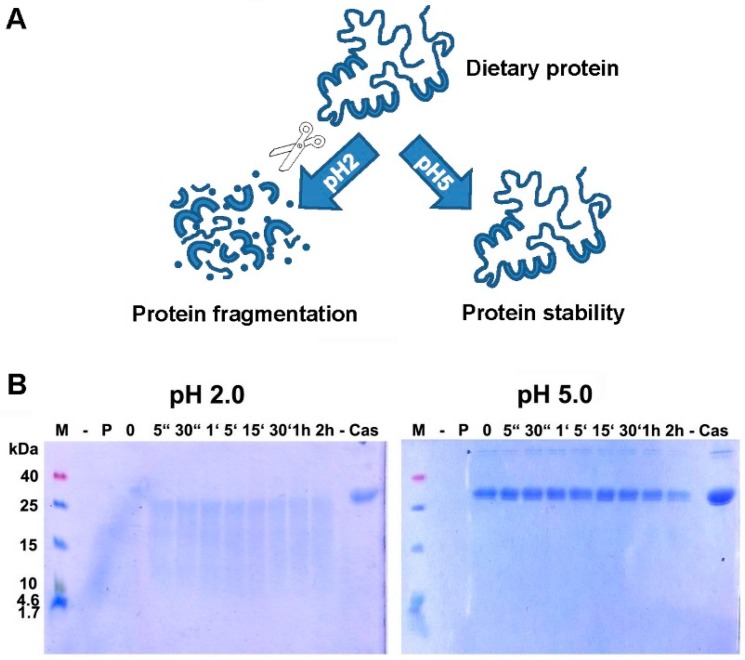
(**A**) Digestion of proteins is hampered when pH increases. Proteins, as part of the daily diet, are digested at low pH and broken down into smaller fragments, whereas a higher pH blocks proper digestion. The resulting bigger fragments or proteins are more easily recognized by the immune system, leading to an increased risk for sensitization or allergic reactions. (**B**) Digestion of α-casein in vitro is hampered when pH increases. Casein was readily broken down by enzymatic digestion with pepsin at pH 2.0, but remained totally intact even after 2 h of incubation with enzyme at pH 5.0. M: molecular weight marker; -: empty lane; P: pepsin; 0: no incubation time, reaction stopped immediately; “: seconds; ‘: minutes; h: hour(s); Cas: casein.

As shown in many previous in vitro experiments, the proper digestion by pepsin is hindered when the pH is increased (Figure 1), and this is true for a number of food proteins, like hazelnut [93], codfish [94], milk [95], and casein (Figure 1).

It is clear that food intake per se changes the gastric pH, which can increase from a median fasting baseline value of pH 1 to pH 4.5 with ingestion of the meal [96]. The buffer capacity thereby depends on the food composition and meal constituents. However, this effect is transient, as ongoing acid production is responsible for a subsequent decrease of the pH, which returns to ca. pH 1 about 260 min after the start of the meal [96]. Applying acid-suppressing substances can disturb this process and induce a long-lasting elevation of the gastric pH up to 5.0 [97].

In a number of food animal models, the effect of this pH-elevation was shown in vivo, as feeding digestion-labile antigen under concomitant acid-suppression resulted in a clear Th2-response and allergy symptoms [98,99,100,101,102,103,104].

This acquired sensitization capacity was true for different proteins, like codfish, hazelnut or ovalbumin, and even oral drugs, in the mouse model [99] and also in humans [105]. Importantly, several types of acid-suppressing or -neutralizing medication, like base powder [106], sucralfate [102], H2-receptor blockers [107] and proton pump inhibitors [101] produced this effect. The outcome of the immune response may depend on timing of the anti-acid drug application in relation to food uptake, and on the dosage of the antigen [101,108].

Gastric acid suppression might further impact on intestinal pH levels and consequently on protein digestion in the intestine [109]. This assumption, however, requires further investigations in clinical settings.

Undoubtedly, knowledge derived from experimental as well as in vitro studies simulating human gastric digestion has to be confirmed using human samples and should be preferentially translated into a clinical setting to confirm the relevance for patients. In 1992, Burks and coauthors reported a 100-fold and 10-fold reduced IgE binding capacity of peanut and soybean allergens, respectively, after exposure to enzymes mimicking human digestion [110]. The different outcome for major food allergen sources was underlined by a study performed more than 10 years later using codfish as a model antigen [111]. After digestion with simulated gastric fluid, the IgE binding capacity of codfish proteins was reduced more than 10,000-fold. This was shown in a reduced histamine release activity from basophil of healthy donors, which were passively sensitized with sera from codfish allergic patients. Also in a clinical setting, the impact of gastric enzymes on fish allergenicity was confirmed [94]. The diameter of positive skin test reactions was significantly reduced after pre-digestion of allergens. Furthermore, the lowest observed adverse effect level in double-blind, placebo controlled food challenges (DBPCFC) was significantly higher. The pre-digestion was performed with gastric enzyme tablets clinically used for patients with reduced gastric acid secretion. Also for celery allergens, the influence of gastric enzymatic digestion on allergenicity could be confirmed in celery allergic patients with a mean age of 72 years [112]. Even in this age group, skin test reactivity was significantly altered when test allergens were pre-incubated with digestive enzymes, highlighting the impact of gastric digestion on food allergenicity.

Deduced from these data, enzymatic hydrolysis of food proteins could help to reduce the IgE-binding capacity and allergenicity in allergic patients. In our group, we could show that insects, which are used as novel food, can be treated with enzymes from the food industry for protein breakdown. The remaining smaller peptides or amino acids from the insect extracts completely lost their cross-recognition of IgE from shrimp- and house dust mite-allergic patients and more important also lost their capacity to elicit positive skin prick test reactions in shrimp-allergic patients [Pali-Schöll et al., MS in revision].

Besides IgE binding, allergenicity has been additionally defined as the capacity of proteins to elicit IgE formation [113]. Based on this definition, not only in situations with already establish food allergy, but also during the development of food adverse reactions, protein degradation might play a major role in the context of allergenicity. As mentioned above also for murine models, interference with gastrointestinal digestion was confirmed to play a major role also in food allergy development. Most studies evaluated situations of impaired gastric acid secretion due to anti-ulcer drug intake. In a first study 152 adult patients being treated for 3 months with either H2-receptor or proton pump inhibitors due to dyspeptic disorders such as reflux, gastritis erosions, or gastric ulcers, were screened for food specific IgE reactivity. A boost of existing IgE or *de novo* IgE formation was found in one fourth of all included patients [95]. In a sub-group of these patients who had developed hazelnut-specific IgE during anti-ulcer treatment, not only could sensitization towards hazelnut be confirmed by specific IgE antibodies and positive skin prick tests: hazelnut allergy was proved in 3 out of 5 patients with elevated hazelnut-specific IgE titers after the 3 months treatment with gastric acid-suppression medication also by positive provocation tests [93]. Moreover, for aged patients living in a geriatric nursing home, the intake of anti-ulcer drugs was found to be associated with a significant shift of the immune response towards a type 2-environment [114]. Not only in elderly, but also in pediatric patients, anti-ulcer drug intake was reported to be associated with the development of food allergy [115,116]. In line, a recent cohort study of 792,130 children demonstrated a higher allergy risk for children being treated with either antibiotics or acid-suppressive medication during the first 6 months of life [7].

Importantly, this influence factor of hindered gastric digestion also seems to play a role during pregnancy, where anti-acid medication of the mother leads to an enhanced risk of asthma or allergy in the offspring in the mouse model [104]. In humans large health register studies and meta-analyses confirmed the increased risk associated with intake of this medication during pregnancy for the development of allergic diseases in children later in life [117,118,119,120], even though prospective studies are missing.

Underlining the role of protein digestion during the sensitization process to food allergen, not only hindrance of digestion due to gastric acid-suppressive medication, but also restriction of digestion due to bariatric gastric bypass surgery might play a fundamental role. To limit the caloric intake of morbidly obese patients, only a small pouch of the stomach remains after surgery interventions such as Roux-en-Y gastric bypass or sleeve gastrectomy [121]. In a pilot study sensitization to an increasing number of common food compounds was detected after gastric bypass surgery [122]. These studies highlight the important role of protein digestibility in the context of allergenicity. However, it is obvious that protein digestion is one of the determinants influencing food allergenicity among others, like protein solubility, size or abundance in a specific food [123].

Thus, it seems to be of special relevance to consider that impaired enzymatic protein digestion is associated with enhanced allergenicity of food proteins. Different mechanisms may be of relevance: (i) a hindered protein digestion through elevated gastric pH or reduced digestive capacity due to bariatric surgery could result in bigger protein fragments that would be recognized by the cells of the immune system; (ii) contained Th2-adjuvants (like aluminum in sucralfate) could direct the immune response towards a Th2-response [100,103], and the allergic milieu could then even be transferred from pregnant/lactating mothers to the offspring [104]; (iii) the dietary content changed during acid-suppression with different remnants ending up in the lower digestive tract could change the composition of the microbiome [98].

## 5. Summary

A number of factors influence the development of food allergies, including the situation in the digestive system. An interference with proper digestion and absorption can be posed by (i) food processing (Maillard reaction, aggregation) [89], (ii) suppression of gastric acid [109,124], (iii) application of adjuvant substances into the digestive tract (aluminum components) [100,103], or (iv) deletion of parts of the digestive system (bariatric surgery) [122]. Several of these processes and factors have been shown to influence the digestive process in vitro, and for some of them the in vivo effect on allergenicity was proven (like for anti-acid drugs). Nevertheless, many knowledge gaps still exist with need for further research studies (see Box 1).

As many of these factors came into play only recently in human evolution, they could probably also explain an important part of the recent increase in prevalence of adverse food reactions.

Box 1 **Knowledge gaps.**

**What Is Well Established?**

**What Should be Further Investigated?**
Amylase action influences the resulting remnants of ingested starch and thereby the microbiomeWhether different amylase action and concentration, e.g., in stress situations, also leads to different outcome regarding allergenicity of the foodFood processing changes protein structure and digestibilityWhether food processing might impact on gastrointestinal pH levelsHeating can lead to glycation and Maillard products, and thereby influences digestibility of involved proteinsWhether proteins become more able to sensitize, or to elicit reactions in allergic patientsAnti-ulcer medication and antacids elevate gastric pH levels and consequently influence food protein digestionWhether gastric acid suppression influences also intestinal pH levels and small intestinal protein digestionLoading of lipid transfer proteins (LTP) with ligands changes their digestibilityWhether loading of LTP changes their immunogenicity and allergenicity in vivoBlocking of gastric digestion increases the risk for allergic sensitizationWhether the subsequent intestinal digestion is also influenced by the changed gastric pH. Whether a functional intestinal digestion could equalize the detrimental sensitizing effect of a blocked gastric digestion

## Abbreviations

AGE	advanced glycated end products
ATI	amylase trypsin inhibitor
DBPCFC	double-blind placebo-controlled food challenge
GIT	gastrointestinal tract
MR	Maillard reaction
MRP	Maillard reaction products
NCGS	non-celiac gluten-sensitivity
OVA	ovalbumin
PC	phosphatidylcholine
PPI	proton pump inhibitor
RDS	rapidly-digestible starch
RS	resistant starch
SDS	slowly-digestible starch
